# Orthopaedic Practice Setting Opinions: Heat Maps for a Hot Topic

**DOI:** 10.2106/JBJS.OA.25.00219

**Published:** 2025-10-22

**Authors:** Zachary N. Jodoin, Daanish Sheikh, Morgan Gable, Tyler Williamson, Steven Shin, Ryan Rose

**Affiliations:** 1 UT Health San Antonio Department of Orthopaedics, San Antonio, Texas; 2 Cedars Sinai Department of Orthopaedics, Los Angeles, California

## Abstract

**Background:**

Selecting an orthopaedic surgery practice setting is a career-altering decision, yet guidance is often anecdotal. Despite existing resources, no consolidated data compare surgeon experiences across practice models. This study surveys orthopaedic surgeons across Texas and California, to quantify perceived strengths and weaknesses of academic, private, hospital-employed, and privademic practice environments.

**Methods:**

An anonymous survey was distributed through professional networks and organizations between May 2024 and May 2025. Respondents identified their current and prior practice settings, subspecialties, and employment ZIP codes. They rated each practice setting in categories including autonomy, salary, ancillary income opportunities, education, research, administrative burden, reputation, community respect, and work-life balance. Responses were aggregated into a heatmap, with subgroup analysis conducted based on employment history and location.

**Results:**

A total of 100 orthopaedic surgeons responded 45% academic, 30% private, 16% hospital-employed, and 8% privademic. Subspecialty distribution was balanced. Academic surgeons rated research opportunities, professional reputation, and continued education highly (p < 0.001), and ancillary income was rated poorly (p < 0.001). Private practitioners valued autonomy, salary, and ancillary income (p < 0.001), but rated research opportunities and continued education poorly (p < 0.001). Hospital-employed surgeons had no categories rated highly. Privademic surgeons had favorable views on autonomy, salary, and income opportunities (p < 0.001), and no categories were rated poorly. Regional comparison showed California surgeons perceived lower academic autonomy (p = 0.048) and work-life balance (p = 0.037), along with less favorable views on salary and income (p = 0.007).

**Conclusions:**

This survey highlights distinct tradeoffs across orthopaedic practice models and locations. Academic models offer professional and educational benefits but are limited in financial upside, whereas private and privademic settings offer enhanced autonomy and compensation. California surgeons reported less favorable perceptions, especially with compensation, highlighting potential regional influences on employment satisfaction. These findings may inform future decisions in orthopaedic career planning and workforce policy.

**Level of Evidence:**

Level IV. See Instructions for Authors for a complete description of levels of evidence.

## Introduction

Choosing a practice setting is crucial for an orthopaedic surgeon's career path. This decision is influenced by various professional opinions and resources^1-4^. The American Academy of Orthopaedic Surgeons (AAOS) offers materials to aid practitioners, including an in-depth guide on subspecialties and practice types^5-8^. These resources allow insight into the ever-changing landscape of orthopaedic practice, but they can be challenging to navigate and do not offer comprehensive guidance.

To date, no consolidation of surgeon practice-setting opinions has been performed. As a result, no objective consensus exists regarding the ideal practice setting based on applicant priorities. Instead, surgeons must make life-altering decisions founded on, oftentimes, a single mentor's subjective experiences and biases^1-4^.

This study surveys the professional opinions of orthopaedic surgeons from across the country employed in all practice setting types. The survey aims to identify what each employment setting prioritizes in various categories, including monetary compensation, professional autonomy, educational opportunities, and work-life balance. By consolidating the opinions of the orthopaedic community, surgeons can gain more granular insight into what to expect from a practice setting when considering employment.

## Methods

### Survey and Data Collection

The survey gathered opinions from orthopaedic surgeons regarding each practice setting's strengths and weaknesses. Current and prior practice settings, subspecialty, and zip code of employment were recorded. The practice environments were defined as academic practice, consisting of training institutions focusing on the education of residents and/or fellows, private practice, including solo or group practices, hospital or health system employment, and a hybrid or “privademic” model, in which surgeons are part of a private group that partakes in high-level surgical education. Questions were structured to solicit quantitative responses in which each practice setting category would rank great, average, or poor based on the survey takers' perception of said category. The categories included practice autonomy, salary, ancillary income opportunities (i.e., surgery center ownership and physical therapy), educational opportunities, research, administrative load, professional reputation-building opportunities, community respect, and work-life balance (Supplemental File 1). The survey was generated and distributed through XM Qualtrics. Responses were collected over 1 year during which participants completed the survey anonymously.

### Sample Recruitment

The survey was distributed to surgeons within Texas and California. The survey was sent out through professional connections and orthopaedic society channels, including the Texas Orthopaedic Association (TOA) and the California Orthopaedic Association (COA). By leveraging personal and professional networks, the survey aimed to capture a wide range of opinions from a large and experienced cohort of surgeons. Texas tort reform, especially the caps on noneconomic and punitive damages, has reduced malpractice lawsuits and lowered liability insurance premiums. These changes have improved physician recruitment and retention by creating a more predictable and favorable legal climate for medical practice^9^. California and Texas were selected primarily for their accessibility through the COA and TOA, with the added advantage of notable differences in tort reform and policy.

### Data Aggregation and Analysis

Survey responses were conglomerated, and the average response for each category was calculated to reflect the population opinion. These data were generated into a heat map to visually represent responses. This method allowed for a comprehensive overview of the general sentiment among orthopaedic surgeons regarding different practice settings and subcategories. Subgroup analysis was conducted for respondents' all-time employment settings. This analysis included only respondents who had been employed in a given setting for more than 1 year, ensuring that opinions reflected the perspectives of surgeons with substantive experience in that practice environment. An additional subgroup analysis was completed comparing Texas and California, to evaluate potential regional disparities.

Heat maps were generated using a 5-tier color system. Dark red represented high dissatisfaction (≥75% of survey responses were poor), light red represented moderate dissatisfaction (the majority, but ≤75%, of survey responses were poor), yellow represented an average or indifferent tone (the majority of survey responses were average), light green represented moderate satisfaction (the majority, but ≤75%, of survey responses were great), and dark green represented high satisfaction (≥75% of survey responses were great).

### Statistical Methods

Baseline demographic and practice characteristics were summarized. The primary outcome was surgeon perception of each practice setting characteristic. Primary predictor variables included practice type, current practice location, and overall surgeon preference. Differences in perceptions across practice settings were analyzed using paired *t*-tests and one-way analysis of variance, as appropriate. A p-value of <0.05 was considered statistically significant, indicating a meaningful difference in opinion between practice settings. All statistical analyses were conducted using SPSS software (version 29.1.1; IBM).

### Ethical and IRB Considerations

The survey was conducted in compliance with ethical standards for research and was Institutional Review Board exempt. Survey participation was voluntary and anonymous.

## Results

**Fig. 1:**
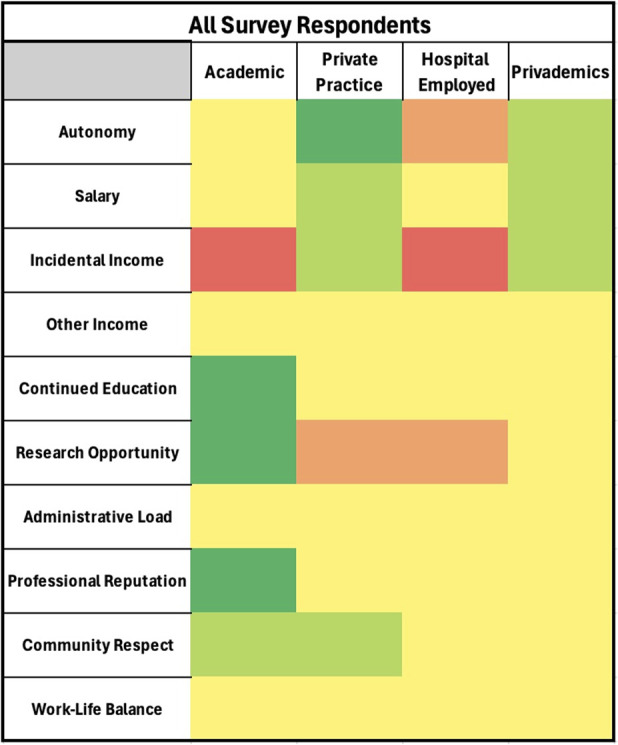
Heat map of all survey respondent conglomerate data. Dark red represents an extremely negative outlook/high dissatisfaction, light red represents moderate dissatisfaction, yellow represents an average or indifferent tone, light green represents moderate satisfaction, and dark green represents extremely positive outlook/high satisfaction.

From May 1, 2024 to June 1, 2025, 100 survey responses were recorded. Forty-five respondents were currently employed in an academic setting (45%), 30 were private (30%), 16 were hospital employed (16%), 8 were privademic (8%), and 1 was retired (1%). Although most respondents were currently academically employed, all-time respondent employment was more representative. Forty-nine respondents were employed in academics for >1 year in their career (49%), 52 experienced private employment (52%), 22 experienced hospital employment (22%), 13 privademic (13%), and 10 with military/federal government background (10%). There was a nationally proportional spread of surgical subspecialties among respondents except for hand/upper extremity, which was slightly more represented among the population (Table I). Survey respondents were located throughout the states of Texas and California in expansive distributions (Fig. 1).

**TABLE I:** Demographics of Survey Results

Characteristic	n (%)
Subspecialty	
Hand/upper extremity	30 (30.6)
Sports	14 (14.3)
Adult reconstruction	6 (6.1)
Foot and ankle	9 (9.2)
Trauma	8 (8.2)
Spine	7 (7.1)
Pediatrics	6 (6.1)
General Orthopaedics	9 (9.2)
Oncology	2 (2.0)
Other (specify below)	7 (7.1)
Current practice type	
Academic	45 (45)
Private	30 (30)
Nonacademic hospital employed	16 (16)
Privademics	8 (8)
Career practice type	
Academic	49 (49.0)
Private	52 (52.0)
Nonacademic hospital employed	22 (22.0)
Privademics	13 (13.3)
Military or government	10 (10.2)
Other	3 (3.1)
Response region	
Texas	47 (47.0)
California	39 (39.0)
Other	14 (14.0)

Survey demographic data include current setting, all-time setting, and subspecialty.

### Overall Cohort

**Fig. 2:**
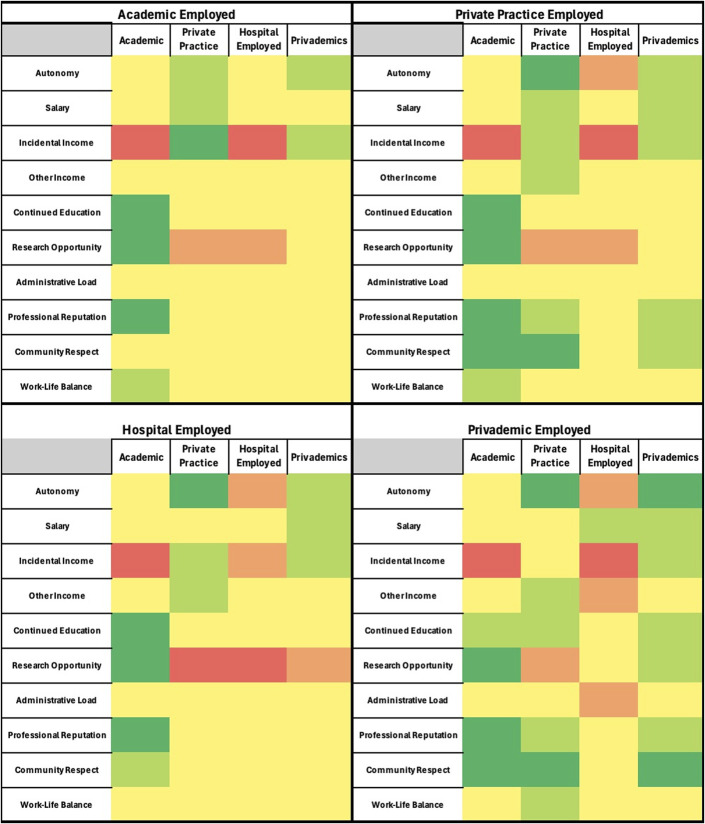
Heat maps of survey responses based on respondent employment settings current of >1 year.

Academic employment had highly rated research opportunities, community respect, professional reputation, and continued education (p < 0.001) (Table II and Fig. 2). It also had a highly valued work-life balance compared with the other employment settings (p = 0.007). Ancillary practice income was perceived as extremely poor (p < 0.001).

**TABLE II:** Consensus Opinion for Each Category and Employment Setting

Factor	Academic	Private	Hospital	Privademic	p-value
Professional autonomy	53%	89%	31%	76%	<0.001
Salary	42%	74%	57%	72%	<0.001
Ancillary income	5%	84%	11%	70%	<0.001
Other income	47%	70%	34%	63%	<0.001
Continued education	90%	43%	51%	60%	<0.001
Research opportunities	92%	14%	28%	53%	<0.001
Administrative load	50%	52%	50%	52%	0.742
Professional reputation	91%	64%	45%	67%	<0.001
Community respect	82%	75%	46%	69%	<0.001
Work-life balance	64%	51%	58%	48%	0.007

Percentages reported are the number of, “Great,” responses. p values were calculated comparing the top and bottom category percentages to reflect difference of opinion for each employment settings.

Private practice employment rated professional autonomy and ancillary income as great (p < 0.001). Salary and community respect were rated highly and considered better compared with academic and hospital employment (p < 0.001). Research opportunities were perceived as extremely limited (p < 0.001). The remaining categories were rated as average.

Hospital employment had no category rated as great. Ancillary practice income was rated as extremely poor (p < 0.001). The remaining categories were rated as average.

Privademics rated ancillary income opportunity, professional autonomy, and salary as great (p < 0.001). No category was rated as poor.

### Employment and Regionality Subgroup Analysis

#### Academic Surgeons

**Fig. 3:**
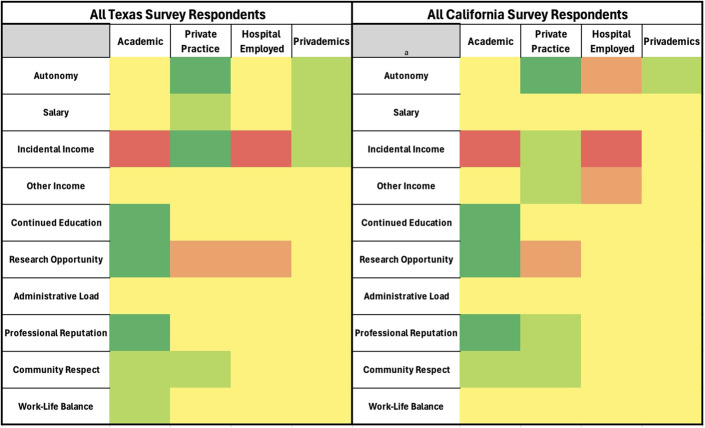
Heat maps of all Texas and California survey respondents.

There was no considerable difference of opinion when comparing academically employed respondents with the overall cohort (Fig. 3).

#### Private Practice Surgeons

Private practice employed respondents had a more positive outlook on community respect and professional reputation across all employment settings except hospital employed, which was still considered average.

#### Hospital/Health System Employed Surgeons

There was no large difference of opinion when comparing hospital-employed respondents with the overall cohort.

#### Privademic Surgeons

Privademic employed respondents had a more positive outlook on community respect and professional reputation across all employment settings except hospital employed, which was still viewed as average. They had an almost universally positive outlook on Privademic employment.

#### Texas vs. California

For most categories, Texas and California responses were similar (Fig. 3). California respondents perceived academic surgeons to have less professional autonomy (60% vs. 50%, p = 0.048) and work-life balance (68% vs. 52%, p = 0.037). California respondents had a largely negative view of salary and ancillary income opportunities for private (p = 0.004), privademic (p < 0.007), and hospital employment (p = 0.045) when compared with Texas.

## Discussion

### Academic Practice

Academic employment was favored for perceived community and interprofessional respect, as well as the ample opportunities for research and education (p < 0.001). The ability to perform high-level research is reflected in the abundant funding available in the academic setting. Eighty-three percent of the National Institutes of Health research budget is granted to academic centers^8^. This allows surgeons to subsidize their productivity and invest freely in research. In addition to participating in research and leading the education of future surgeons, many academic orthopaedic surgeons also take on leadership roles in national organizations and meetings. These responsibilities bring a strong sense of community and professional prestige^10,11^. Almost all respondents agreed that academic employment sacrifices ancillary income opportunities, however (p < 0.001). These opportunities are often constrained by institutional policies or faculty contracts. This can severely limit the ability to passively expand income (Fig. 1).

### Private Practice

The primary benefit of private practice was the unmatched professional autonomy (p < 0.001). Self-employment is associated with higher job satisfaction due to minimized oversight and increased flexibility^12-14^. Private practice was also associated with the most ancillary income opportunity. More than 40% of orthopaedic surgeons report engaging in supplemental income activities beyond their primary employment^15,16^. As reimbursement rates continue to drop, this ancillary income will likely play a vital role in employment decisions in the future^17,18^. A modestly positive outlook on salary potential was reflected in the data, likely attributable to the productivity collections-based compensation model commonly found in the private practice setting. This structure enables surgeons to influence their earnings directly, with income determined by individual workload and motivation^19^. Private practice received negative feedback regarding research opportunities. Time devoted to research often detracts from clinical productivity, which can adversely affect the surgeon's and practice's financial performance. This concern is reflected in the survey's more pessimistic stance related to research engagement (Fig. 1).

### Hospital/Health System Employed Practice

Hospital employment revealed negative views pertaining to ancillary income, autonomy, and research opportunities (p < 0.001). This likely reflects the prioritization of clinical-productivity and health-system financial goals. Recent data show a growing trend toward hospital employment among orthopaedic surgeons. Twenty-one percent of young surgeons are employed by hospitals, compared with just 12% of their senior counterparts^2^. This may be support utilization of hospital employment as a steppingstone because young surgeons develop their geographic and practice preferences. There has also been a significant trend in vertical and horizontal consolidation of orthopaedic practices over the past decade, leading to further hospital employment and affiliations^20^. Notably, survey responses revealed no positive feedback in any category related to hospital employment, raising potential concerns about the future trajectory of the field. Most survey categories, however, received average ratings, suggesting a neutral stance toward hospital employment among orthopaedic surgeons. Shifting priorities toward financial stability and risk aversion over traditional drivers of career satisfaction such as autonomy, academic engagement, and unconstrained income potential may be occurring (Fig. 1).

### Privademic Practice

The responses for privademics revealed a striking balance between academic and private practice responses, reflecting the hybrid employment structure. All feedback was largely positive or average, with minimal negative responses, suggesting that this model may appeal to individuals seeking a balance between academic and clinical work or those uncertain about their career path. However, this employment model can be rare because it was the least represented within the survey population at just 8% (Fig. 1).

### All-Time Employment Analysis

Academic and hospital-employed surgeons showed opinions consistent with the overall cohort. Private practice surgeons reported a more favorable view of community respect and professional reputation across all settings except hospital employment, which may further reflect the disdain for this employment setting. Similarly, privademic surgeons had a generally positive outlook on all settings—especially their own—with hospital employment again viewed less favorably. Notably, the privademic group had only 13 respondents, which likely limits the generalizability (Fig. 2).

### California vs Texas

We consider this study to be nationally representative, considering the responses were from 2 of the most populous and demographically diverse states, California and Texas, each representing opposing ends of the health care and policy spectrum. California has embraced Medicaid expansion and broader public health infrastructure, while Texas maintains the highest uninsured rate in the nation^21-23^. These differences make the 2 states ideal comparators to assess regional influences on orthopaedic surgeon experiences.

The California cohort's negative perception of income in primarily private and privademic practice (p < 0.001) likely reflects the increasing financial burdens specific to the state. Over the past few decades, rising overhead, administrative complexity, and malpractice insurance have significantly reduced profit margins in private practice. These challenges, compounded by California's high cost of living, taxes, and inflation, contribute to a more pessimistic outlook on income potential with higher financial risk. By contrast, this effect appears less pronounced in Texas, where the cost of living and administrative overhead are lower^24^. The nuanced response variances may be helpful in further tailoring these data toward state and population-specific employment (Fig. 3).

### Limitations

This survey study has several limitations. It was conducted among orthopaedic surgeons practicing in Texas and California, which may introduce regional bias and limit national generalizability. Expanding the study to a broader geographic population would help capture a more representative sample. In addition, most respondents were currently employed in academic settings, potentially skewing the data toward academic viewpoints. This limitation is partially mitigated because most academic respondents reported previous experience in private or alternative practice models. There were also a disproportionate number of upper extremity surgeons among respondents, although representation across other subspecialties was otherwise well-balanced.

## Conclusion

Existing data from the AAOS outline employment trends but offer little explanation for the motivations behind said trends. This survey-based study addresses that gap by capturing a broad spectrum of professional perspectives across practice settings. For residents, fellows, and established surgeons considering a career change, these findings provide an unbiased guide to the strengths and drawbacks associated with each practice setting type. As the field continues to shift, these data will potentially assist surgeons in making more informed decisions, allowing sustainable career choices that align with their personal and professional goals.

## Appendix

Supporting material provided by the authors is posted with the online version of this article as a data supplement at jbjs.org (http://links.lww.com/JBJSOA/A965). This content was not copyedited or verified by JBJS.
